# Microseismic energy distribution and impact risk analysis of complex heterogeneous spatial evolution of extra-thick layered strata

**DOI:** 10.1038/s41598-022-14538-7

**Published:** 2022-06-27

**Authors:** Xingping Lai, Chong Jia, Feng Cui, Jianqiang Chen, Yupu Zhou, Ganggui Feng, Yuanjiang Gao

**Affiliations:** 1 College of Energy Engineering, School of Energy Resources, Xi’an University of Science and Technology, Xi’an, 710054 China; 2 State Key Laboratory of Coal Green and Safety Development in West China, Xi’an, 710054 China; 3 State Energy Group, Xinjiang Energy Co., Ltd., Urumqi, 830002 China

**Keywords:** Environmental impact, Solid Earth sciences, Mineralogy, Petrology

## Abstract

In the process of deep mining of coal resources, coal seams with better geological conditions are gradually mined preferentially, and the safe and efficient mining of working face in complex and heterogeneous spaces of residual coal seams is an urgent problem to be solved.. Based on the Kuangou Coal Mine as the background, using microseismic monitoring instruments and pressure sensor monitoring systems, the rock pressure appearance and microseismic energy characteristics accompanying the evolution of the overburden strata structure in the mining of solid coal and the lower working face of the gob are analyzed. Research on the precursory characteristics and early warning of micro-earthquakes. The research results show that: (1) The period of the W1123 working face mining under solid coal is relatively frequent, and the energy of microseismic events is higher than that under the mined-out area. However, the overlying rock structure under the gob is loose, broken and easy to move, showing obvious "high frequency-low energy" characteristics. (2) Extremely low values of the number and energy of microseismic events occurrs in the first 3 to 5 days of the rockburst event in the working face, and the locations of the rockburst disaster in the mine were generally distributed at the edge of the low-density area of the microseismic event. The accuracy of rockburst prediction is effectively improved through multi-parameter comprehensive early warning. (3) Roof deep hole blasting and roof cutting pressure relief weaken the roof energy accumulation and the concentrated release of rock formation energy, reduce the roof activity intensity in the microseismic event gathering area, and reduce the occurrence of large-energy events, which will easily induce large shock hazards. The energy event weakens into a slow release of multiple small energy events. This research provides a reference for the safe and efficient mining of working faces in complex space environment.

## Introduction

With the continuous increase of mining depth and mining intensity of coal mines in our country, the geological conditions and mining layout are becoming more and more complicated, which makes the mine mining face increasing the risk of rock burst disasters. Scholars pay more and more attention to the frequent stope dynamic disasters. Due to many factors affecting the occurrence of rockburst, the cognition of the mechanism and prevention mechanism of rock burst needs to be further improved^1–4^. Therefore, it is urgent to establish an effective microseismic monitoring and early warning method for mine mining in complex heterogeneous space, and to carry out the microseismic energy distribution and shock risk analysis of the evolution of heterogeneous space, so as to meet the requirements of reducing the risk of rockburst and effective prevention and control of rock burst.

In the process of deformation and failure of coal and rock mass, the generation and expansion of cracks release energy in the form of stress waves, resulting in microseismic events. Many scholars have made attempts in the early warning of rock burst based on the characteristics of microseismic. Among them, Zhenhua WU et al.^5^ obtained the time-series regularity of the precursory information of rockburst microseismic in the graben tectonic region through analysis. The characteristics of MS waves obtained by Xuelong Li et al.^6^ can effectively identify and extract valuable precursor information for rock dynamic disaster prediction. Xianghui TIAN et al.^7^ proposed a new method for early warning of shock hazards based on the maximum daily microseismic energy and the total number of high values of microseismic energy-frequency deviation. Dazhao Song et al.^8^ calculated the relationship between the initial strength of periodic cracks and the stress set according to the Griffith energy theory, and established the criterion for rock burst occurrence. Linming Dou et al.^9^ established the MS multi-parameter index system for dynamic failure of coal and rock, and estimated the critical value of each index. In addition to its application in mine production, microseismic monitoring has also been widely used in the analysis of space–time analysis of seismic zones, thrust earthquakes caused by plates, failure and evolution of faults, slope stability, and tunnel rock bursts^10–15^.

In the research of microseismic caused by mining in working face, Xingping Lai et al.^16,17^ revealed the loss of overlying rock through the characteristics of microseismic "energy-frequency" distribution, cumulative apparent volume, energy index and microseismic *b* value. Precursor characteristics of stability failure. Petr Konicek et al.^18^ implemented monitoring of stress changes and induced seismic activity during longwall mining in complex spaces, forming the application of decompression blasting technology for safe mining of working faces. Xiating Feng et al.^19^ established a dynamic early warning system based on the evolution of microseismic events, which reduced the risk of rock bursts occurring immediately during the excavation of deep-buried hard rock tunnels. At the same time, the application of methods such as seismic energy attenuation coefficient, microseismic event trend, algorithm evaluation of microseismic data, and microseismic full waveform inversion effectively meet the requirements of safe mining in complex spaces^20–23^. In addition, the empirical scaling law is used to estimate the seismic waveform of the epicenter, and the traditional algorithm is used to train the historical monitoring data. According to the strain energy density theory, the regional disintegration energy damage criterion based on the strain gradient is established, which provides effective prediction of mine rock burst and other disasters^24–27^.

The above scholars have made a lot of analysis on the mine-site microseismic monitoring and rock burst occurrence law, and have achieved good application results in practice. However, about solid coal and gob, there is relatively few research on overburden evolution induced by mining in complex space face and impact risk caused by microseismic energy change. For this reason, this paper takes physical coal mining in Kuangou Coal Mine and the working face under the gob as the background to carry out a physical similarity simulation experiment. Combined with microseismic monitoring system and pressure sensor, the distribution law of microseismic events and rock pressure law of solid coal and mined coal rock mass under gob are studied. From the point of view of the accumulation and release of microseismic energy, the rockburst variation law of the working face induced by the complex spatial variation of the extra-thick layered strata is discussed. It provides a scientific basis for the mining and prevention and control of the working face under the complex heterogeneous space of the extra-thick layered strata.

## Engineering background

**Figure 1:**
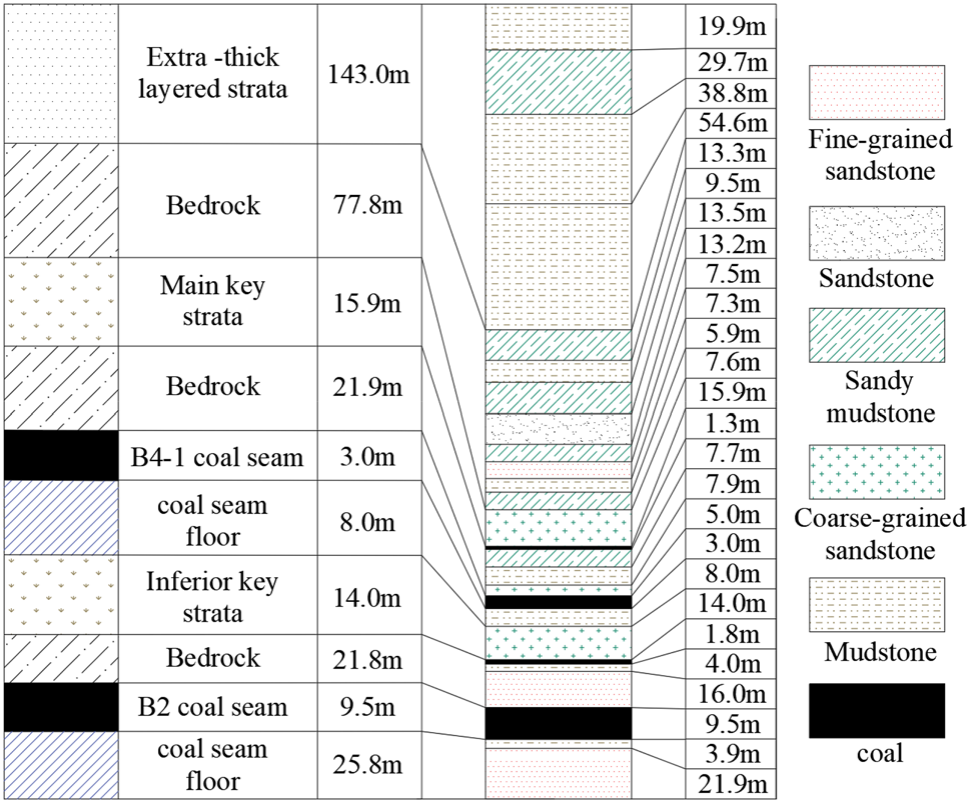
Schematic diagram of stratigraphic structure.

The Kuangou Coal Mine is located in Changji Prefecture, Xinjiang, China. The west wing of the first mining area of the mine mainly mines the B4-1 coal seam and the B2 coal seam, and currently mines the B2 coal seam W1123 working face. The schematic diagram of stratigraphic structure is shown in Fig. 1. The mining depth of W1123 working face is about 392 m, the strike length is 1468 m, and the inclination length is 192 m. The mine is currently mining B2 coal seam with an average thickness of 9.5 m. It adopts fully mechanized caving mining. The roof of the working face is a hard roof, and the crack joints between the coal seam and the roof are not developed and have a tendency to impact. The 15.9 m thick coarse-grained sandstone at the position of 21.9 m above the B4-1 coal seam is the main key layer of overlying strata, which is calculated by the key layer discrimination method^28^. The coarse-grained sandstone at the position of 21.8 m above the B2 coal seam is the sub-key layer of overburden. The stratum with a thickness of more than 18 m is regarded as a extra-thick strata, while there are four obvious layered strata with a thickness of more than 18 m in the overlying strata of the coal seam. Therefore, in this paper, the four strata are regarded as extra-thick layered strata, and the accumulated thickness of the extra-thick layered strata above them reaches 143.0 m. After the W1145 working face of the B4-1 coal seam is mined, the W1123 working face has a process of gradually advancing from the B4-1 solid coal to the W1145 working face gob.

**Figure 2:**
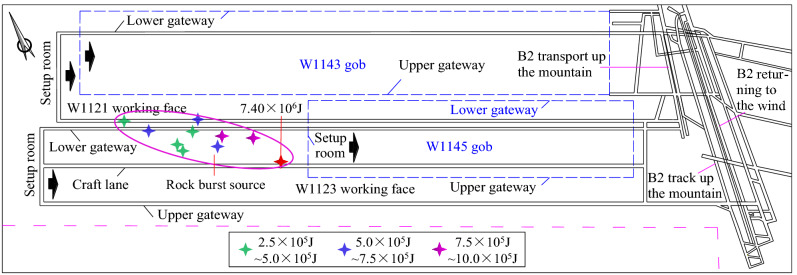
The layout of the working face and the spatial distribution of the previous shock events.

Because of the impact tendency of B2 coal seam and its roof and floor in Kuangou Coal Mine, when W1123 working face approaches and crosses under the gob of B4-1 coal seam, some areas will be affected by the superposition of the upper gob and its own advanced abutment pressure. The movement and collapse of key strata of overlying strata in the mining process of W1123 working face will also affect the production of working face, which will easily lead to the occurrence of dynamic disasters. Figure 2 shows the layout of the working face and the spatial distribution of previous impact events. Previous impact events mainly occurred on both sides of the transportation roadway, that is, the coal pillar and the coal wall near the transportation roadway within 20–50 m. Among them, in the process of W1123 working face gradually advancing to the lower part of gob of W1145 working face, the energy of previous impact events showed an obvious increasing trend, and the maximum energy of impact events reached 7.40 × 10^6^ J.

## Analysis of physical simulation experiment in complex heterogeneous space

### Model design and construction

**Figure 3:**
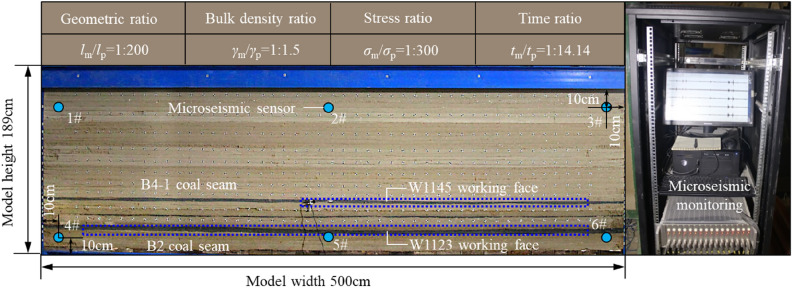
Physical material model construction and microseismic monitoring arrangement.

Based on the geological conditions of W1123 working face in Kuangou Coal Mine, a physical similar material simulation model was built. The experiment adopts a plane strain model frame of 5.0 × 0.3 × 2 m, and the geometric similarity ratio (model: prototype) of the simulation experiment is 1: 200.The paving size of similar experimental model is 5.0 × 0.3 × 1.89 m. Loading a layer of iron brick on the top of the model is equivalent to 40 m thick overburden, and the loading stress is 0.8 MPa. The physical material model construction and microseismic monitoring arrangement are shown in Fig. 3. The physical W1123 working face of the model, after 3 cm open-cut, can achieve the simulation effect of the actual 4.8 m/d advancing speed of the mine as much as possible with a single mining of 2.4 cm. At the same time, the ground pressure phenomenon and microseismic energy evolution law associated with rock breaking are analyzed by floor pressure monitoring and microseismic monitoring.

The simulation material ratio of the physical model is shown in Table 1. Based on the overburden lithology of B2 coal seam proved by ZK201 borehole histogram of W1123 working face in Kuangou Coal Mine, the similar material ratio is formulated. The materials used in the model paving process are: river sand, large white powder, plaster of Paris and water, as well as fly ash that should be added when proportioning coal seams. In this experiment, the composition and strength of similar materials similar to from the actual ones, which can simulate the actual rock strata better.

**Table 1:** Simulation material ratio of physical model.

Rock stratum	Actual thickness/m	Simulated thickness/cm	Serial number	River sand/kg	Plaster/kg	Big white powder/kg	Packing thickness and times (cm)	
A	29.2	14.6	837	42.67	1.60	3.73	2	8
B	11	5.5	737	23.10	0.99	2.31	1.1	10
A	48	24	837	42.67	1.60	3.73	2	12
A	42	21	837	64.00	2.40	5.60	3	7
A	50	25	837	42.67	1.60	3.73	2	13
C	13	6.5	828	27.73	0.69	2.77	1.3	5
A	10	5	837	21.33	0.80	1.87	1	5
C	14	7	828	21.33	0.53	2.13	1	7
D	13	6.5	928	28.08	0.62	2.50	1.3	5
C	8	4	828	21.33	0.53	2.13	1	4
B	7	3.5	737	37.80	1.62	3.78	1.8	2
A	6	3	837	21.33	0.80	1.87	1	3
C	8	4	828	21.33	0.53	2.13	1	4
E	16	8	728	84.00	2.40	9.60	4	2
C	9	4.5	828	32.00	0.80	3.20	1.5	3
A	8	4	837	21.33	0.80	1.87	1	4
E	5	2.5	728	21.00	0.60	2.40	1	3
B4-1 coal	3	1.5	20:20:1:5	15.65	0.78	3.91	1.5	1
A	8	4	837	21.33	0.80	1.87	1	4
E	14	7	728	73.50	2.10	8.40	3.5	2
B3 coal	1.8	0.9	20:20:1:5	10.43	0.52	2.61	1.8	1
A	3	1.5	837	32.00	0.80	3.20	1.5	1
B	16	8	737	84.00	3.60	8.40	4	2
B2 coal	9.5	4.75	20:20:1:5	10.43	0.52	2.61	9.5	1
A	4	2	837	42.67	1.60	3.73	2	1
B	22	11	737	42.00	1.80	4.20	2	6

A-mudstone, B-fine-grained sandstone, C-sandy mudstone, D-sandstone, E-coarse-grained sandstone.

### Evolution law of complex spatial overlying strata structure

The evolution characteristics of different overlying strata structures in the mining process of the working face cause the microseismic difference changes in the mining process of the working face. In order to clarifying the microseismic change characteristics caused by the mining process of the working face, this paper firstly analyzes the evolution law of overlying strata structures in the mining process of the working face.

**Figure 4:**
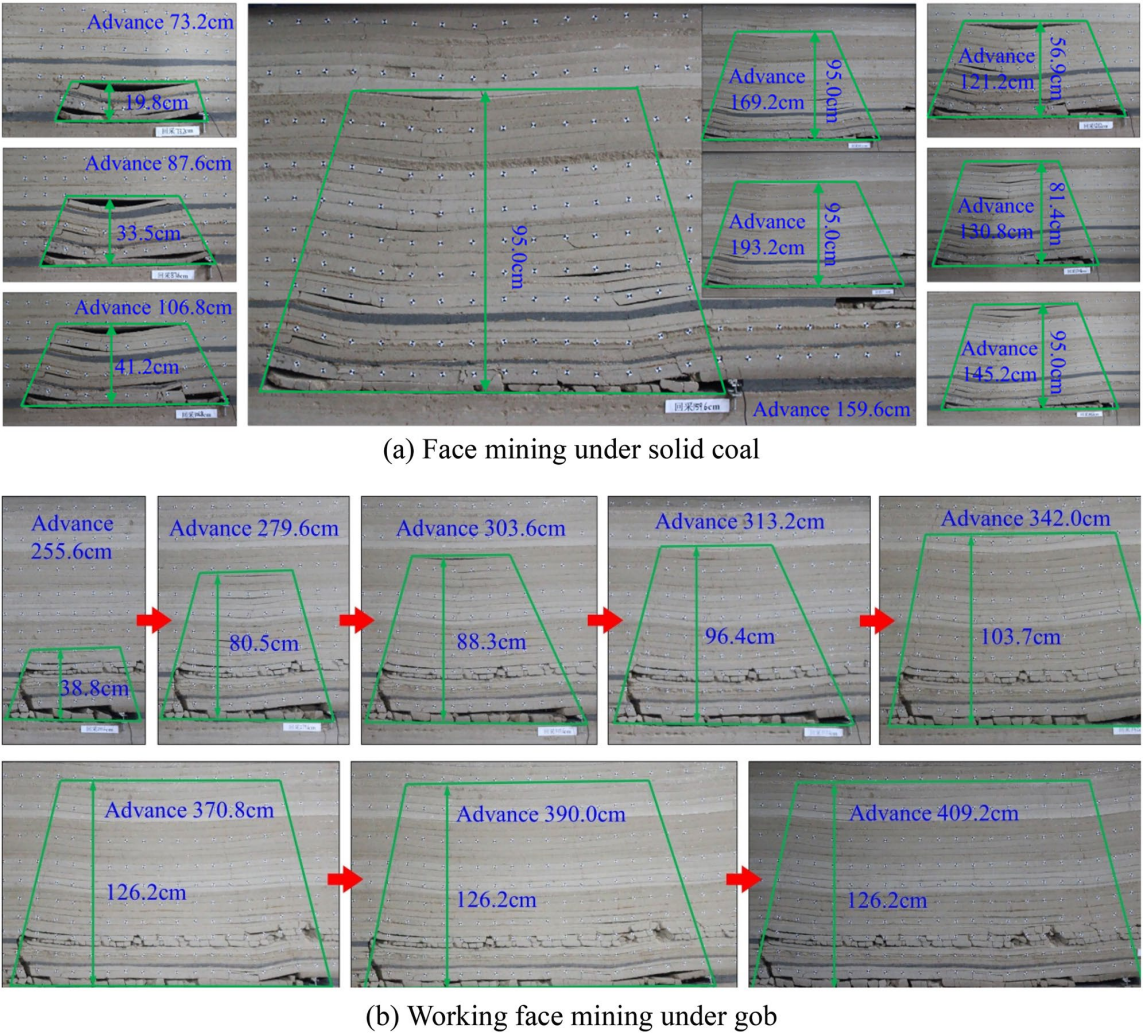
Overburden evolution characteristics of partial periodic weighting process in W1123 working face mining.

The evolution characteristics of overlying strata in partial periodic weighting process of W1123 working face mining are shown in Fig. 4, in which Fig. 4a and b respectively show the evolution characteristics of overlying strata structure in working face mining under solid coal and gob. When the working face under the solid coal advances by 73.2 cm, the overlying strata on the upper part of the working face collapse for the first time, and an obvious broken area with a height of 19.8 cm is formed. During the first caving of the working face to the advancing of the working face to 145.2 m, with the mining of the working face, the breaking height of overlying strata gradually increased. Among them, when advancing toward the working face by 87.6 cm, 106.8 cm, 121.2 cm and 130.8 cm, the overburden failure is 33.5 cm, 41.2 cm, 56.9 cm and 81.4 cm respectively. When the working face advances to 145.2, when mining under the solid coal, the overlying strata breaking area is constantly moving forward with the advancing of the working face, but the height of the breaking area is relatively stable, with the breaking height of 95.0 cm. When the working face under the gob advances by 73.2 cm, the overlying strata on the upper part of the working face collapse intensively, and an obvious broken area with a height of 38.8 cm is formed. In the process of the working face under the gob gradually advancing from 198.0 cm to 370.8 cm, the broken height of overlying strata gradually increases with the mining of the working face. Among them, when advancing toward the working face by 279.6 cm, 303.6 cm, 313.2 cm and 342.0 cm, the overburden failure is 80.5 cm, 88.3 cm, 96.4 cm and 103.7 cm respectively. When the working face is advanced to 370.8 and then mined under the gob, the broken area of overlying strata is constantly moving forward with the advancement of the working face, but the height of the broken area is relatively stable, with the breaking height of 126.2 cm.

**Figure 5:**
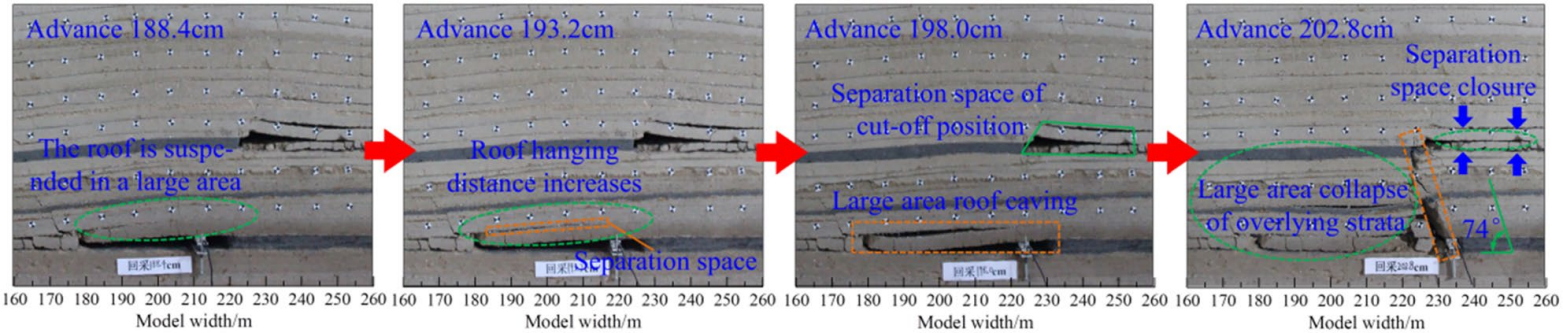
Evolution characteristics of overlying strata in mining face from solid coal to critical position of gob.

The evolution characteristics of overlying strata in working face mining from solid coal to the critical position of gob are shown in Fig. 5. When the working face advances by 188.4 cm, the roof of the working face hangs in a large area and there is obvious separation space, and the overlying strata on the upper part of the roof are relatively complete. When the working face advances by 193.2 cm, the working face is located below the open cut of W1145 working face, and the hanging distance of the working face roof increases, which significantly increases the separation space. When the working face is advanced by 198.0 cm, the hanging distance of the working face roof reaches the limit value, and the roof is broken in a large area with a breaking distance of about 35.2 cm. When the working face is advanced by 202.8 cm, the upper part of the working face is hung in a wide range. Under the action of large-scale deflection and extrusion of overlying strata, the overlying strata of the upper part of the working face under the solid coal caving in a large area, and the overlying strata breaking angle of 74 is formed. Under the influence of extrusion, the separation space of the gob at the cut-off position of the upper coal seam working face is closed.

### Energy evolution characteristics of complex spatial microseisms

Microseismic monitoring can realize the time, space and energy monitoring of coal and rock fracture events in the mining process of working face, and it is a necessary monitoring means for rock burst mines. Through the statistical analysis of microseismic event data in the mining process of W1123 working face, we can analyze the quantity and energy of microseismic events in different stages of mining in complex heterogeneous space, and support the effective verification of safe mining in working face.

**Figure 6:**
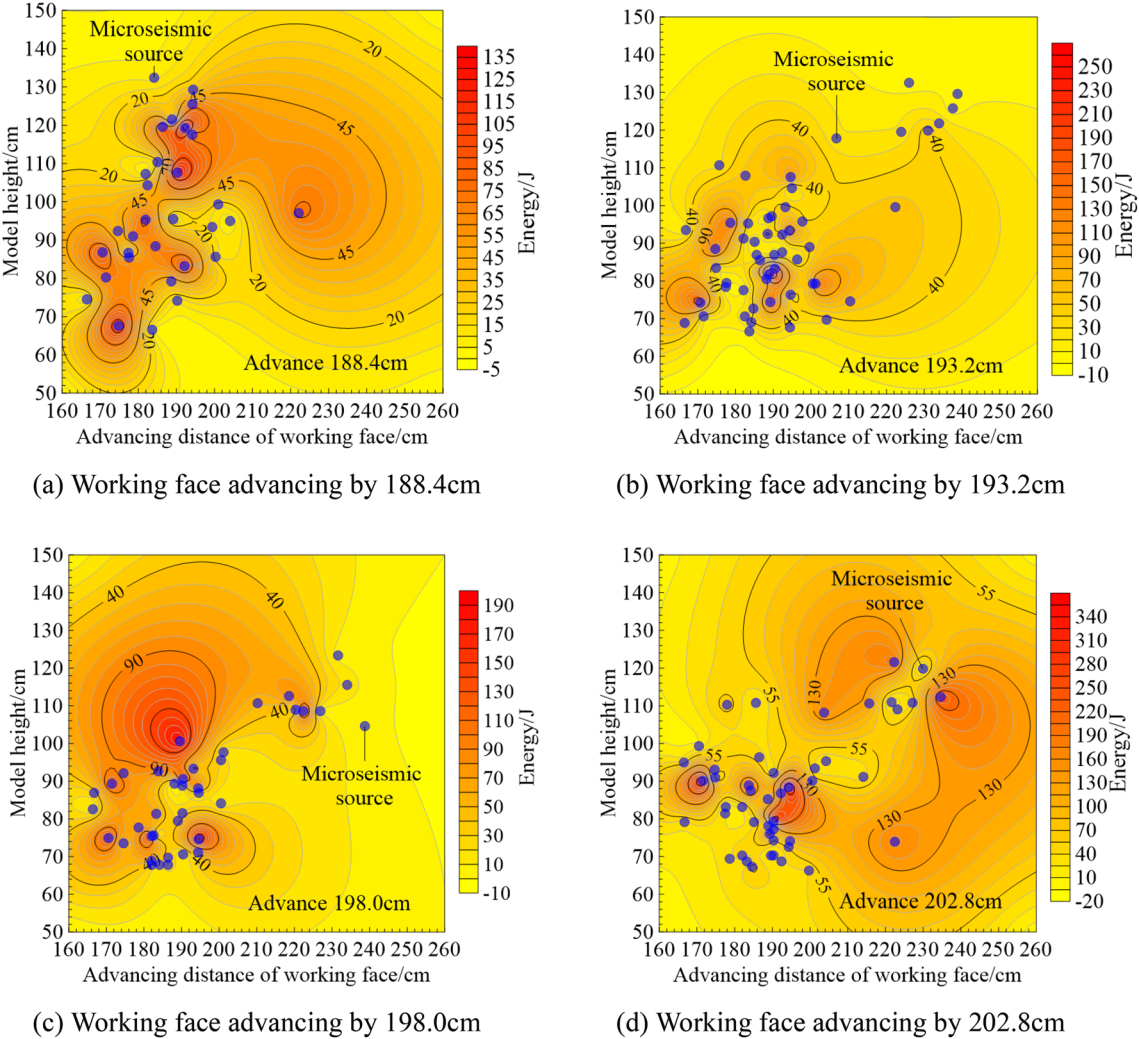
Microseismic energy distribution nephogram of mining face from solid coal to critical position of gob.

Figure 6 shows the microseismic energy distribution cloud map of the mining face from solid coal to the critical position of the gob, in which Fig. 6a–d are cloud map of microseismic energy distribution when the working faces respectively advancing to 188.4 cm, 193.2 cm, 198.0 cm and 202.8 cm. When the working face advances by 188.4 cm, the microseismic energy is mainly located in the range of 166.3–195.2 cm in the advancing direction of the working face. There is an obvious large energy distribution area within the model height range of 106.6–123.5 cm, and the maximum energy in the area reaches 135 J. When the working face advances 193.2 cm, the microseismic energy is mainly located in the range of 166.3–204.3 cm in the advancing direction of the working face. There is an obvious large-energy event at the model height of 75.3 m, where the energy reaches 250 J. When the working face advances 198.0 cm, the microseismic energy is mainly located in the range of 167.8–202.6 cm in the working face advancing direction, and there is an obvious large-energy event at the model height of 99.7 m, where the energy reaches 190 J. When the working face is advanced by 202.8 cm, there are two obvious large energy accumulation areas in the range of 188.5–198.7 cm in the advancing direction of the working face and the model height of 79.2–89.3 cm. The energy is about 370 J.

Comparing the evolution of the overburden and its microseismic energy distribution of the mining face in Figs. 5 and 6, it can be seen that the evolution of the overburden structure during the advancing process of the working face causes different microseismic energy variation characteristics. When the working face advances to 188.4 cm, the change of the roof of the working face is small, and the upper overlying rock is relatively complete, so the microseismic energy generated by the propulsion process of the working face is small, and the energy value of the energy accumulation area is low. When the working face gradually advanced to 193.2 cm and 198.0 cm, the overlying rock changed significantly, and the roof separation space gradually developed and formed a large area of roof collapse, which made the energy value of the upper overlying rock energy accumulation area high. 135 J when the working face is advanced to 188.4 cm. When the working face is advanced to 202.8 cm, the central strata between the coal seam groups are broken in a concentrated manner, generating high-energy microseismic energy, and the energy value of the accumulation area reaches 370 J. With the closure of the abscission space at the incision position of the W1145 working face, there is a certain activation effect of overlying rock in the upper space in the range of 215.3–234.5 cm in the forward direction of the surface, which is accompanied by the generation of higher energy.

**Figure 7:**
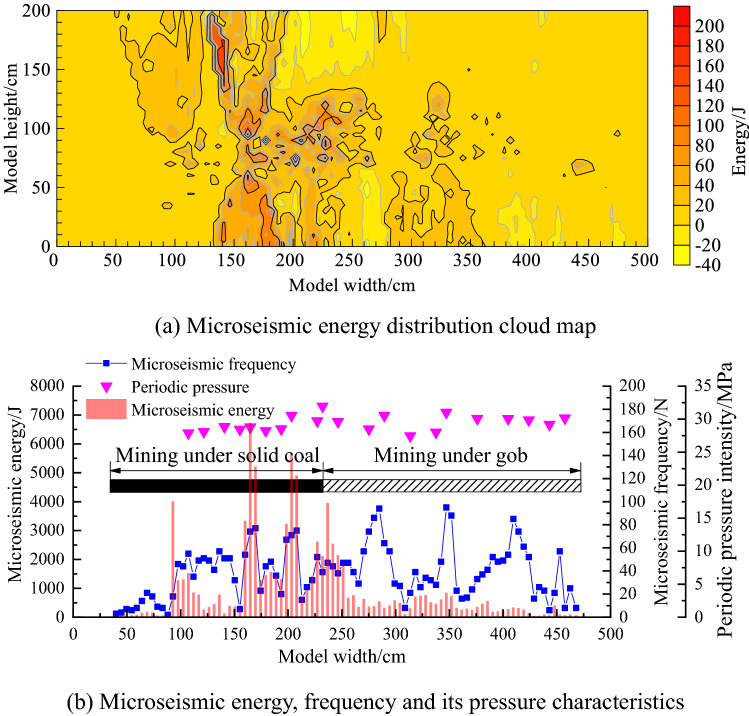
Microseismic energy distribution and pressure characteristics of W1123 working face during the whole mining process.

The energy and frequency of microseismic events can reflect the change of coal and rock stress. The higher the microseismic event energy and the more frequent the vibration, the greater the stress concentration of coal and rock mass and the more serious the damage. Therefore, the microseismic energy distribution and its weighting characteristics in the whole mining process of W1123 working face are drawn based on the microseismic and support pressure monitoring data, as shown in Fig. 7, in which Fig. 7a is the microseismic energy distribution cloud map, and Fig. 7b is the microseismic energy, frequency and weighting characteristics.

It can be seen from Fig. 7a that the microseismic energy distribution cloud map shows that the key layer of solid coal is relatively complete and can better support the concentrated effect of the upper overburden compared with the key layer of the broken overburden in the gob. The severity of overburden damage in the mining process of working face 132.5–163.3 is obviously greater than that in other areas. During the mining process of the W1123 working face, the microseismic energy is mainly located in the model width of 132.5–163.3 cm, and its energy is mostly in the range of 140–220 J. It can be seen from Fig. 7b that the microseismic energy, frequency and the characteristics of the incoming pressure show that the microseismic and large energy events generated by the overburden fracture of the W1123 working face are mainly concentrated in the mining process of the working face under the solid coal. The energy peak positions of the four microseismic events generated by mining under the solid coal face are located at the model widths of 92.8 cm, 164.8 cm, 230.2 cm and 227.2 cm, respectively. When the working face advances to these four positions, the roof will periodically fracture and collapse when the elastic energy accumulated by the upper overburden reaches its ultimate strength, releasing the accumulated elastic energy. It is easy to induce rock burst. However, the energy value of W1145 gob is relatively small during mining, and the energy of microseismic events is mostly between 0 and 50 J. The energy of microseismic events under solid coal is higher than that under gob, and the frequency of microseismic events under gob is higher than that under solid coal mining.

Table 2 shows the statistical table of the periodic back pressure data of the W1123 working face mining. The W1123 working face will form different back pressure characteristics when mining the solid coal of the upper B4-1 coal seam and the mined-out area. Combining with the position of the periodic pressure in Fig. 7b, it can be seen that the initial pressure occurs at 73.2 cm below the solid coal, and the recovery occurs at 73.2–193.2 cm, with a total of 8 periodic pressures. In the 193.2–432 cm mining under the gob, there are 12 times of cyclic pressure, and the cycle of mining under solid coal is relatively frequent. The overlying rock structure under the gob is loose, broken and easy to move, and the arched structure is damaged and the supporting capacity is weakened, so the accumulation and release period of the microseismic energy is short. The fluctuation of the cyclic pressure value under the gob is small, and the support bears more overlying rock. Therefore, the cyclic pressure value of the W1123 working face in the mining process under the gob is slightly higher than that under the solid coal.

**Table 2:** Statistical table of the period to pressure data of W1123 working face mining.

Under solid coal			Under the gob		
Number of times/N	Advance distance/cm	Bracket pressure /MPa	Number of times/N	Advance distance/cm	Bracket pressure/MPa
1	73.2	27.89	1	198	31.95
2	87.6	28.18	2	212.4	29.62
3	106.8	28.85	3	241.2	28.49
4	121.2	28.44	4	255.6	30.53
5	130.8	28.80	5	279.6	27.46
6	145.2	28.23	6	303.6	28.01
7	159.6	28.52	7	313.2	30.01
8	169.2	30.53	8	342	30.10
9	193.2	29.72	9	370.8	30.08
			10	390	29.86
			11	409.2	29.19
			12	423.6	30.17

During the mining process of W1123 working face, a total of 21 obvious cycles were formed.

## Precursor characteristics and comprehensive early warning of rock burst micro-earthquakes

### Time–space precursory distribution characteristics of microseisms occurring in rock burst

**Figure 8:**
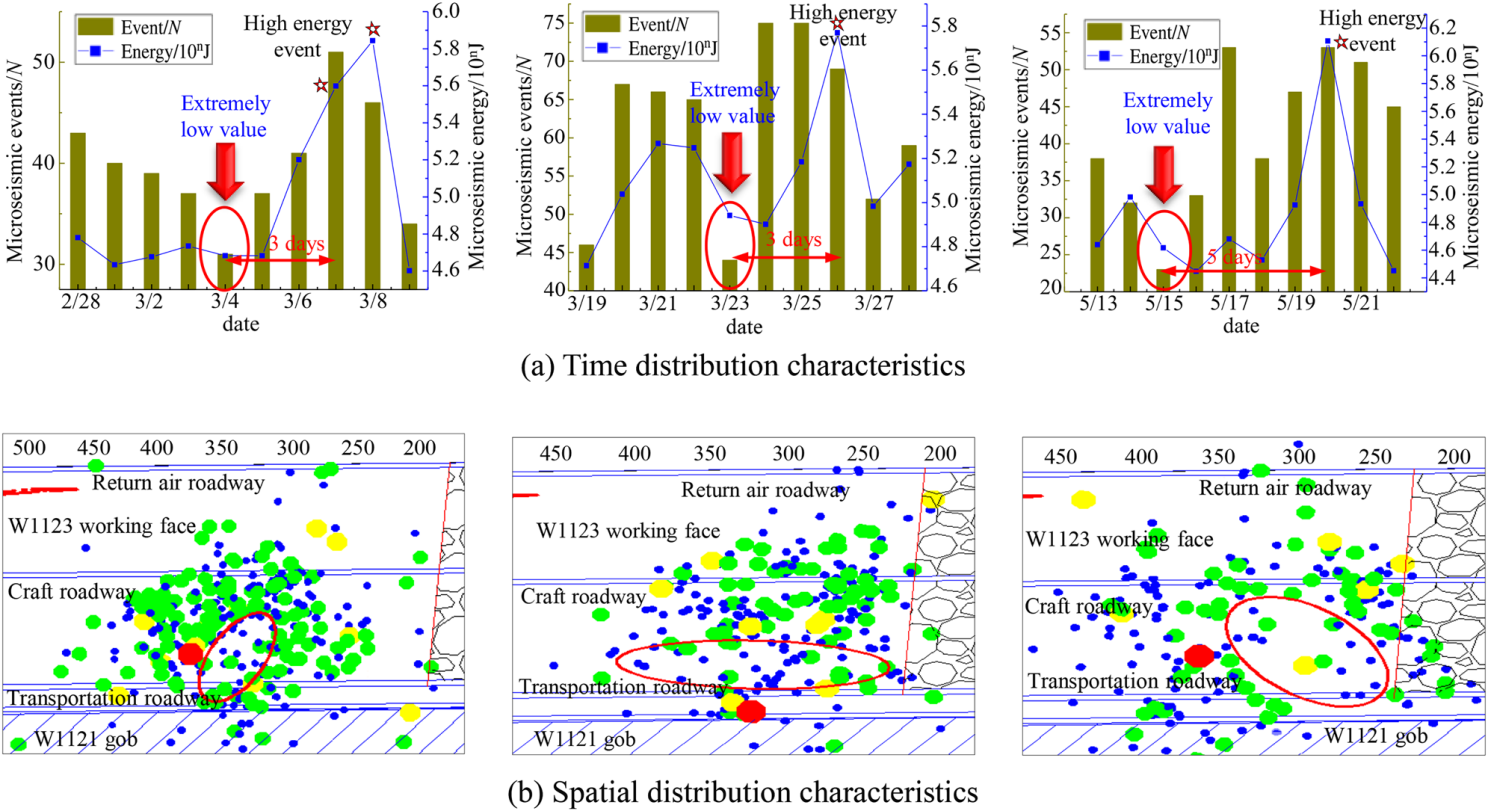
Temporal-spatial precursor distribution characteristics of shock microseisms.

According to the real-time monitoring of microseisms on site, the temporal-spatial precursor distribution characteristics of microseisms occurring in the face impact are drawn as shown in Fig. 8. From the time distribution characteristics of microseismic occurrences in Fig. 8a, it can be seen that the single occurrence of shock-hazardous high-energy events. The daily microseismic energy is generally in the range of 10^5.6^ to 10^6.2^ J. At the same time, the number and energy of microseismic events are extremely low in the first 3 to 5 days before the rock burst event occurs on the working face. Taking March 4 as an example, there were 32 microseismic events in a single day, which was significantly smaller than the 52 events when the large-energy event occurred three days later.

Figure 8b shows the spatial distribution characteristics of microseisms that occurred in the impact, and it can be seen that there is an obvious low-density area of microseismic events near the location where the rock burst occurs in the middle and lower parts of the working face, and the location of rock burst disasters in the mine is generally distributed in the microseismic area, that is the edge of the low-density area of the event. Before the rock burst occurs in the mine, the low-density areas with the spatial distribution of microseismic characteristics are consistent with the phenomenon of lack of earthquakes, so the phenomenon of low-density areas with the spatial distribution of microseisms is used as an early warning indicator for rock burst on the working face.

### *B*-value early warning index based on G-R relation

The magnitude-frequency relation of earthquakes is called G-R relation, and the distribution characteristics of microseismic energy-frequency when rock burst occurs accord with G-R relation. Therefore, the *b* value of G-R relation is taken as the early warning index, and a method of obtaining the early warning value of the early warning index of rock burst precursors by dynamic change rate and critical value method is proposed. According to the principle of least square method, the formula of *b* value^29^ is:

1 $$ b = \left\{ \begin{gathered} \frac{{\sum\nolimits_{i = 1}^{m} {\lg E_{i} \sum\nolimits_{i = 1}^{m} {\lg N_{i} - m\sum\nolimits_{i = 1}^{m} {\lg E_{i} \lg N_{i} } } } }}{{m\sum\nolimits_{i = 1}^{m} {\lg^{2} E_{i} - (\sum\nolimits_{i = 1}^{m} {\lg E_{i} } )^{2} } }}(N_{i} \ne 0,and \, not \, all \, 1) \\ 0(N_{i} { = }0,or \, all \, 1) \\ \end{gathered} \right. $$

In the formula, *m* is the total number of energy bins; *E*_i_ is the energy of the ith grade; *N*_i_ is the actual number of events of the ith grade of energy.

**Figure 9:**
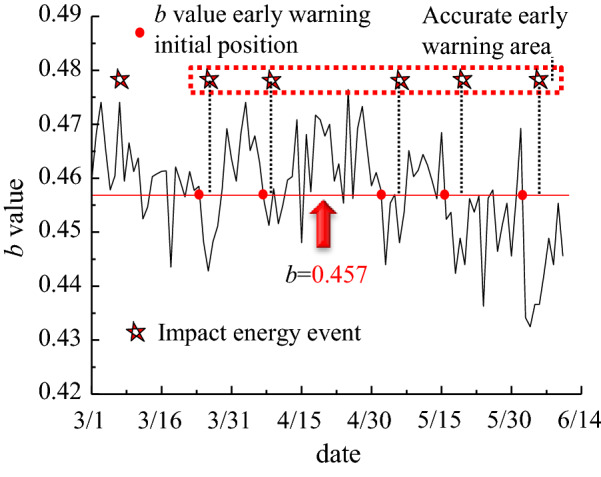
Distribution and variation characteristics of b value.

Figure 9 shows the distribution of b value and its variation characteristics based on the microseismic monitoring data. The average value of *b* value is used as an early warning indicator. When the *b* value is less than 0.457, the early warning of rock burst is carried out. The initial forecast time of the *b* value early warning index is 2–7 days earlier than the occurrence time of the rock burst, and the early warning method is timely; the probability of using the *b* value to accurately warn the rock burst is 83.3%, therefore the *b* value early warning method can achieve effective early warning.

### Comprehensive early warning of rock burst based on microseismic monitoring

**Figure 10:**
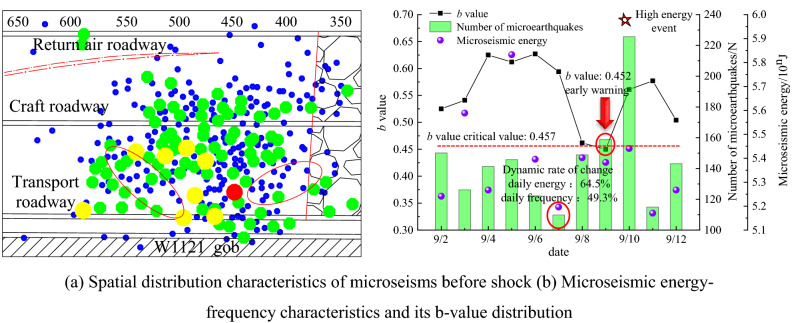
Comprehensive early warning results of rock burst.

Through the comprehensive analysis of the microseismic spatial distribution characteristics, the microseismic energy-frequency distribution characteristics, and the b value distribution before the shock occurs, a multi-parameter comprehensive early warning method for rock burst is established. The comprehensive early warning results of rock burst are shown in Fig. 10. From Fig. 10a the spatial distribution characteristics of microseisms before the shock occurred, it can be seen that low-density areas appeared in the middle and lower parts of the working face within 4 days before the shock occurred.

From Fig. 10b microseismic energy-frequency characteristics and its *b* value distribution, it can be seen that the dynamic change rates of daily energy and frequency in the three days before the shock occurred were 64.5% and 49.3%, respectively. According to the distribution of its *b* value, it can be known that the *b* value is less than the early warning critical value of 0.457 of the day before the occurrence of rock burst. The effect of multi-parameter comprehensive early warning has strong complementarity. Through the establishment of the multi-parameter comprehensive early warning method of rock burst, the joint early warning can effectively improve the accuracy of rock burst forecast.

Through the real-time monitoring and analysis of microseismic data, the mine rock burst is well warned, but the missed rock burst will still cause damage. In order to fundamentally prevent rock burst, combined with the analysis of microseismic change law caused by the evolution of overlying strata structure above, the pressure relief measures of roof blasting and roof cutting are adopted, and the microseismic results are verified on site.

## Analysis of pressure relief effect of heterogeneous space roof

In the mining process of W1123 working face, the hard roof and extra-thick strata can't collapse in time, which leads to large-scale roof hanging in the gob, extremely high stress concentration and energy accumulation in front of the working face, which makes it easy to induce huge energy and vibration load when the direct roof breaks in front of the working face. Therefore, after obtaining the distribution characteristics of microseismic precursors in time–space and the *b*-value early warning index of G-R relationship, and knowing the location of the complex heterogeneous space where there is a risk of impact, this chapter considers reducing the mine pressure intensity of the working face from the source, and implementing deep-hole blasting and roof cutting to relieve pressure on the thick and hard direct roof and key strata within a certain range of the advanced working face, weakening the strength of hard rock strata, reducing the hanging roof area of goaf and the breaking distance of key strata, and reducing the energy accumulation of the roof of the advanced working face, so as to ensure the safety of the working face.

### Working face roof pressure relief scheme

**Figure 11:**
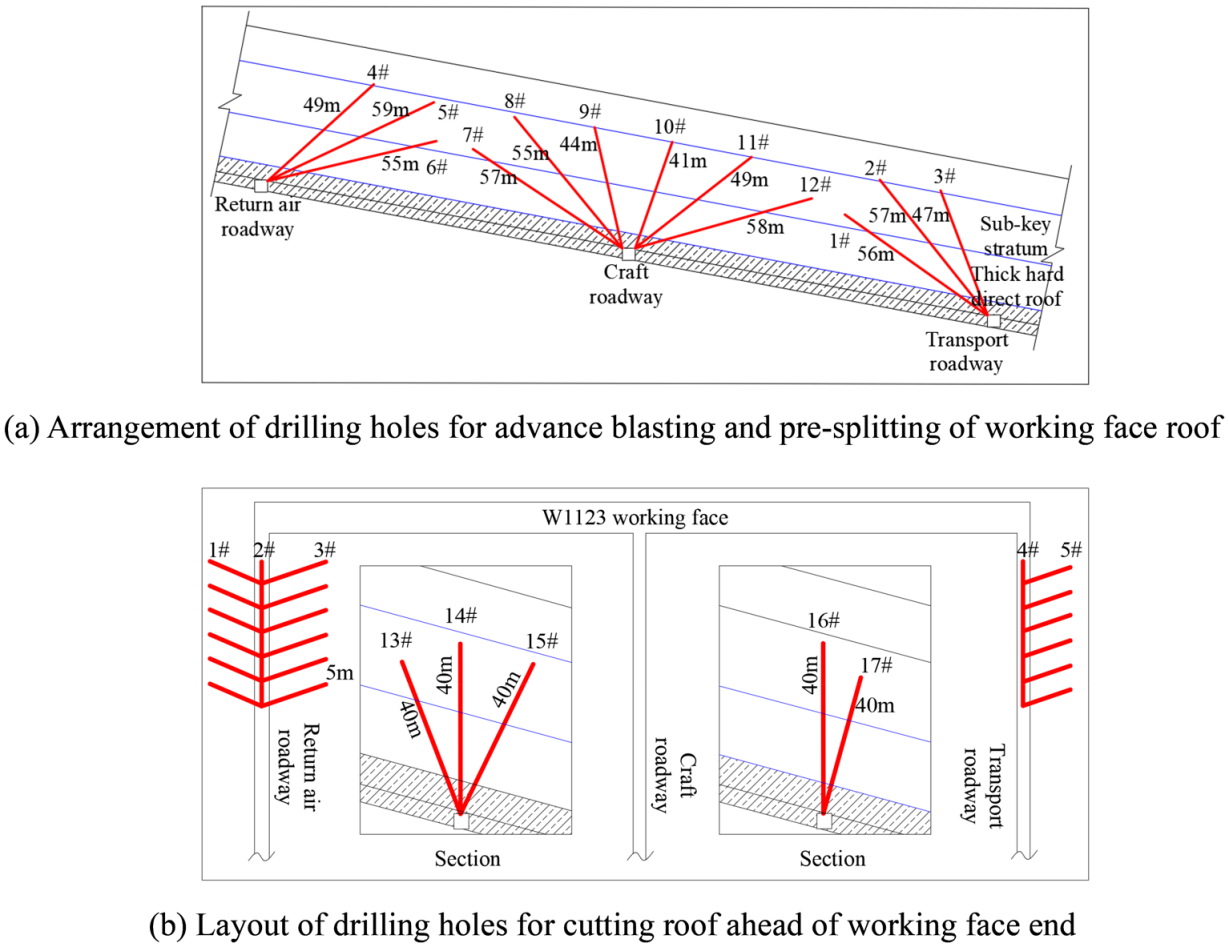
Pressure relief scheme of working face roof and its upper and lower ends.

The energy accumulation area of the W1123 working face when the hard roof is broken and unstable is mostly located within 50 m in front of the advanced working face. For this reason, the hard roof within 50 m of the advanced working face is blasted to reduce the stress concentration and energy accumulation of the roof in this area. The thick and hard direct roof and key layers of the working face are not easy to collapse. Combined with the feasibility of roof blasting, economic requirements and control effects, the thick and hard direct and sub-key layers of the working face are blasted. The blasting height is about 44.3 m, and the roof of the working face is blasted. The Pressure relief scheme of working face roof and its upper and lower ends is shown in Fig. 11.

Figure 11a shows the advance pre-splitting drilling arrangement in the roof of the working face. According to the calculation of the blasthole spacing in the group, a group of blastholes is arranged every 10 m along the working face strike. Along the inclination, each group of blastholes is arranged at different angles along the upper and lower troughs and the craft roadway, to ensure that the roof pre-blasting is completed 50 m ahead of the working face. In order to ensure that the working face can meet the requirements of the mining and evaporating roof during the normal mining period, and avoid the over-long end lane leading to the timely release of the elastic performance of the roof, the top of the working face is cut in advance to relieve pressure, and its upper and lower ends are advanced. The top cut hole arrangement is shown in Fig. 11b. According to the actual engineering conditions that the hard roof of the W1145 and W1123 working faces of Kuangou Coal Mine is difficult to collapse, the fracture angle of the key layer is taken as the basis for designing the elevation angle of the advanced pressure relief drilling. The advanced blasting of the working face and its top and bottom cutting parameters are shown in Table 3.

**Table 3:** The advanced blasting of the working face and its top and bottom cutting parameters.

Location	Hole number	Borehole length/m	Elevation angle/°	Charge length/m	Sealing length/m	Charge/kg
Advance blasting hole of transportation roadway	1	46	40	31	15	78
	2	47	56	32	15	80
	3	37	73	24	13	60
Advance blasting hole of return air roadway	4	49	42	36	13	130
	5	59	25	43	16	165
	6	55	12	40	15	150
Advance blasting hole of technical roadway	7	57	38	42	15	160
	8	55	55	40	25	150
	9	44	81	32	12	110
	10	41	68	31	10	105
	11	49	37	36	13	130
	12	58	14	42	16	160
The end of return air roadway and transportation roadway cut the top hole in advance	13	40	67	30	10	100
	14	40	90	30	10	100
	15	40	62	30	10	100
	16	40	80	30	10	50
	17	40	80	30	10	50

### Microseismic characteristics of roof before and after pressure relief

**Figure 12:**
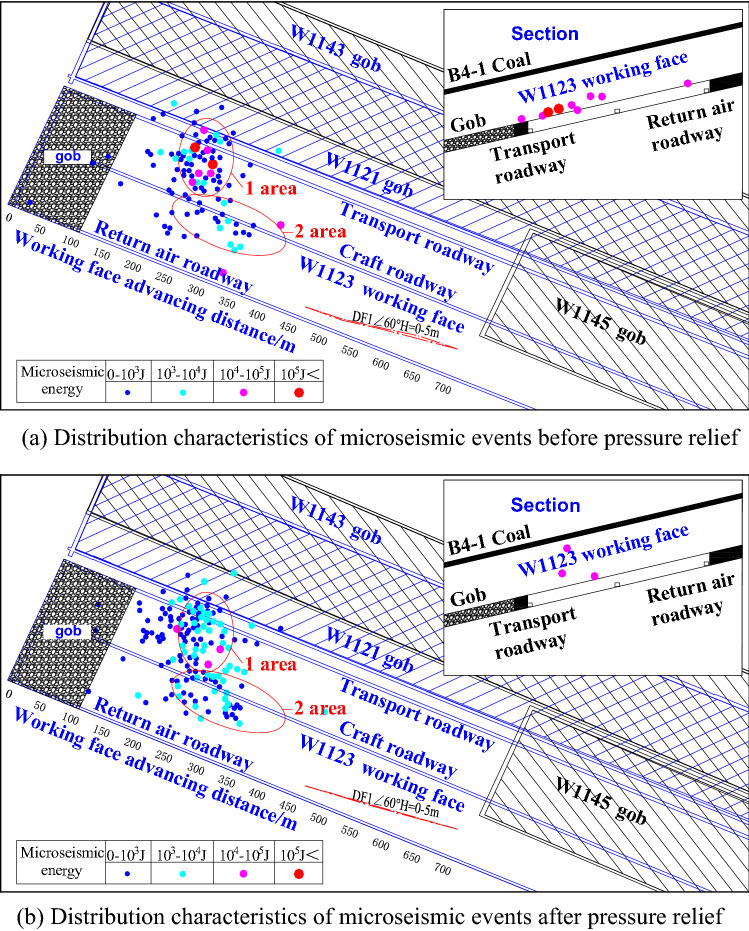
Distribution characteristics of roof microseismic events before and after pressure relief.

According to the changes of microseismic events before and after pressure relief by advanced blasting and roof cutting, the distribution characteristics of roof microseismic events before and after pressure relief are drawn as shown in Fig. 12. The distribution characteristics of microseismic events before pressure relief are shown in Fig. 12a. The roof microseismic events mainly occur in the area of Area 1 and Area 2 in front of the working face. Before blasting, the roof microseismic events in the blasting area have no aggregation phenomenon, but the energy value of the microseismic events is larger. The distribution characteristics of microseismic events after pressure relief are shown in Fig. 12b. There is no obvious change in the concentrated area of microseismic events after blasting. The microseismic events with higher energy are obviously reduced in Zone 1 and Zone 2, and the microseismic events with lower energy are obviously increased. There are 9 obvious high-energy seismic events near the roof of the working face before blasting. After blasting, the microseismic events with energy greater than 10^4^ J are greatly reduced, and the microseismic events are obviously transferred to the upper part of the working face.

The total microseismic energy before and after pressure relief is 4.25 × 10^5^ J and 2.87 × 10^5^ J respectively, and the energy after pressure relief decreases by 32.47%. Deep-hole roof blasting and roof cutting and pressure relief weaken the roof energy accumulation and concentrated release of rock energy, reduce the roof activity intensity in the microseismic event gathering area, reduce the occurrence of high-energy events, increase the crack expansion of roof rock strata, effectively weaken the high-energy events that are easy to induce impact danger into multiple low-energy events and slowly release them, effectively satisfying the safe and efficient mining of working face in complex space environment.

## Conclusions

1. The evolution of overlying strata structure in heterogeneous space from solid coal to the critical position of gob in W1123 working face is relatively severe. Under the action of large-scale deflection and extrusion of overlying strata, the upper strata collapse intensively, and an overlying strata breaking angle of 74 is formed. The periodic weighting of mining under solid coal is relatively frequent, and its microseismic event energy is higher than that under gob. However, the mining overburden structure under gob is loose, broken and easy to move, and its microseismic energy accumulation and release period is short, and microseismic energy shows obvious characteristics of "high frequency-low energy".
2. Extremely low values of microseismic events and energy occurred in the first 3 to 5 days before rock burst occurred in the working face, and the locations of rock burst disasters in mines were generally distributed at the edge of low-density areas of microseismic events. Before rock burst occurred, the low-density area appeared in the middle and lower part of the working face within 4 days, and the *b* value was less than the warning critical value of 0.457. Multi-parameter comprehensive early warning of microseismic spatial distribution characteristics, microseismic energy-frequency distribution characteristics, *b* value distribution, etc. can effectively improve the accuracy of rock burst prediction.
3. Roof deep-hole blasting and roof cutting and pressure relief weakened roof energy accumulation and concentrated release of rock energy, reduced roof activity intensity of microseismic event gathering area, reduced occurrence of high-energy events, increased crack expansion of roof rock strata, effectively weakened high-energy events that easily induced impact risk into multiple low-energy events and slowly released them, which effectively satisfied the safe and efficient mining of working face in complex space environment.

## Supplementary Information

Supplementary Information.

## Data Availability

All the data supporting this work were collected at the similar simulation platform of Xi'an University of Science and Technology in China and Xinjiang Energy Co., Ltd., State Energy Group. All the data provided in this study can be obtained from the newsletter author.
