# Supplementary material for: Microseismic energy distribution and impact risk analysis of complex heterogeneous spatial evolution of extra-thick layered strata
Source: Sci Rep. 2022 Jun 27;12:10832. doi: 10.1038/s41598-022-14538-7 (PMC9237134; doi:10.1038/s41598-022-14538-7)
Supplement: Supplementary file 1 — Supplementary Information. [file 41598_2022_14538_MOESM1_ESM.docx]

Supplementary material

The main datasets generated and analyzed during the current study are provided in the "Figures" file, some raw data are not made public due to [data non-disclosure reasons], but are available from the corresponding authors upon reasonable request.

Note: Figure 1a, Figure 2-Figure 5 in the "Figures" file can be directly opened by "PPT", Figure 6 and Figure 7a can be directly opened by "surfer", Figure 7b can be directly opened by "origin", Figure 1b and Figure 11-12 can be directly opened by " CAD" opens directly.
